# The cerebellum and fear extinction: evidence from rodent and human studies

**DOI:** 10.3389/fnsys.2023.1166166

**Published:** 2023-04-21

**Authors:** Alice Doubliez, Enzo Nio, Fernando Senovilla-Sanz, Vasiliki Spatharioti, Richard Apps, Dagmar Timmann, Charlotte L. Lawrenson

**Affiliations:** ^1^Department of Neurology, Center for Translational Neuro- and Behavioral Sciences (C-TNBS), Essen University Hospital, University of Duisburg-Essen, Essen, Germany; ^2^School of Physiology, Pharmacology and Neuroscience, University of Bristol, Bristol, United Kingdom

**Keywords:** cerebellum, fear extinction, fMRI, prediction error, fear behaviour, cerebro-cerebellar circuits, electrophysiology

## Abstract

The role of the cerebellum in emotional control has gained increasing interest, with studies showing it is involved in fear learning and memory in both humans and rodents. This review will focus on the contributions of the cerebellum to the extinction of learned fear responses. Extinction of fearful memories is critical for adaptive behaviour, and is clinically relevant to anxiety disorders such as post-traumatic stress disorder, in which deficits in extinction processes are thought to occur. We present evidence that supports cerebellar involvement in fear extinction, from rodent studies that investigate molecular mechanisms and functional connectivity with other brain regions of the known fear extinction network, to fMRI studies in humans. This evidence is considered in relation to the theoretical framework that the cerebellum is involved in the formation and updating of internal models of the inner and outer world by detecting errors between predicted and actual outcomes. In the case of fear conditioning, these internal models are thought to predict the occurrence of an aversive unconditioned stimulus (US), and when the aversive US is unexpectedly omitted during extinction learning the cerebellum uses prediction errors to update the internal model. Differences between human and rodent studies are highlighted to help inform future work.

## Introduction

Learned fear is a key survival response, but successfully extinguishing fear when the threat is no longer present is equally important for behavioural adaptation. It is thought that an inability to extinguish fearful memories underlies anxiety disorders such as post-traumatic stress disorder (Milad and Quirk, 2012). All animals (including humans) have a variety of defensive responses to a threat. While humans can be interviewed about their emotional feeling of fear towards a threat, defensive behaviours such as flight/freezing are often used as a proxy for the fear state in rodents and, as a result, caution is needed in such an assignment. Under experimental conditions, defensive responses can be elicited using fear conditioning paradigms which provide a model in both humans and other species to study “fear” over time. Defensive responses can be triggered not only by the adverse stimulus itself, but also by an initially neutral stimulus predicting the occurrence of an adverse event. Pavlovian fear conditioning paradigms use this characteristic to study behavioural and physiological mechanisms involved in fear acquisition and extinction. During fear acquisition, an unconditioned stimulus (US), inducing innate fear, is repeatedly paired with a conditioned neutral stimulus (CS) to elicit fearful behavioural responses, and so the CS becomes the CS+. To investigate extinction of a fearful memory, the experimenter can observe the conditioned responses when the US is no longer paired with the CS. As extinction training progresses, CS+ related behavioural conditioned responses gradually decrease due to the unexpected omission of the aversive US. Over repeated presentations of the CS alone the omission of the aversive US leads to prediction of the absence of the US, termed extinction learning. This can be compared over time with a CS of similar intensity that has not been paired, termed a CS−.

The contribution of the cerebellum to the emotional network has been described since the late 1930s (Clark, 1939; Zanchetti and Zoccolini, 1954; Berman et al., 1974), however, more recently, it has gained increasing interest in relation to conditioned fear behaviour (Sacchetti et al., 2002; Frontera et al., 2020; Vaaga et al., 2020; Lawrenson et al., 2022). Previous studies have found that the cerebellum is involved in the extinction of learned motor responses (Ernst et al., 2017), raising the possibility that the cerebellum may be supporting a similar role in the extinction of learned emotional responses. This review focuses on evidence for such a role in conditioned fear extinction, from rodent studies that investigate cerebellar functional connectivity with other brain regions and underlying molecular mechanisms, to fMRI studies in humans.

## The neurocircuitry underpinning the cerebellum in adaptive fear behaviours

The neurocircuitry and regional activation underpinning conditioned fear learning and memory has been mapped extensively in rodents (Apps and Strata, 2015; Tovote et al., 2015) and humans (Fullana et al., 2016; Greco and Liberzon, 2016). The cerebellum has been found to play a role in motor (Koutsikou et al., 2014), autonomic (Supple and Leaton, 1990), emotional and cognitive (Timmann et al., 2010; Apps and Strata, 2015; Guell et al., 2018) functions relating to conditioned fear learning (Leaton, 2003; Strata, 2015). Broadly speaking the cerebellum is widely thought to act as, or is part of, an internal predictive system, implicated in associative appetitive and fear learning processes driven by prediction error corresponding to the discrepancy between predicted and actual outcomes (Popa and Ebner, 2019). Even though its role is not fully understood, a summary of its functional and structural connectivity with vital components of the limbic system have previously been highlighted by Apps and Strata (2015). The cerebellum is connected directly with the periaqueductal grey (Teune et al., 2000; Koutsikou et al., 2014; Frontera et al., 2020; Vaaga et al., 2020; Lawrenson et al., 2022), the ventral tegmental area (VTA; Watabe-Uchida et al., 2012; Carta et al., 2019; Pisano et al., 2021), the thalamus (Watabe-Uchida et al., 2012; Apps and Strata, 2015; Gornati et al., 2018; Pisano et al., 2021) and hypothalamus (Dietrichs and Haines, 1989; Apps and Strata, 2015) as well as indirectly with other cortical and subcortical structures such as the amygdala (Jung et al., 2022), the anterior cingulate cortex, hippocampus and the striatum (Moreno-Rius, 2018).

## The cerebellar-periaqueductal grey pathway and fear extinction: rodent studies

**FIGURE 1:**
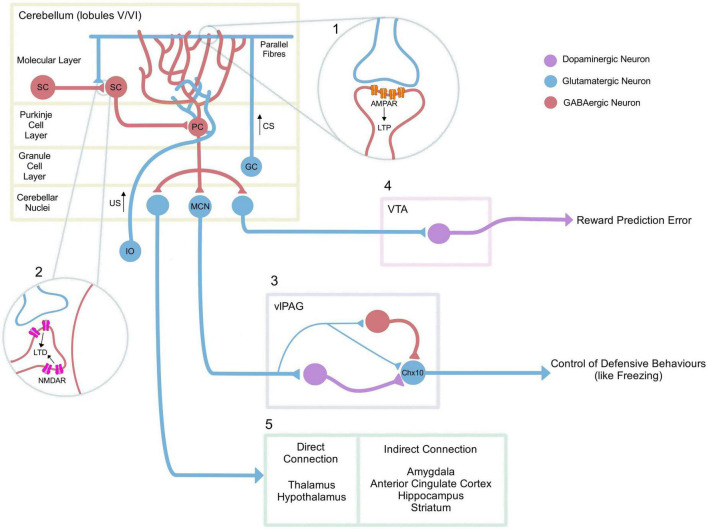
Neurocircuitry underlying adaptive cerebellar fear processes. In classical pavlovian conditioning, the inferior olive (IO) signals the US, while the mossy fibre-parallel fibre pathway (MF-PF) signals the unconditional stimulus. (1) During the acquisition of a fear memory, there is an upregulation of AMPA receptors at the PF-PC synapse, resulting in post-synaptic long-term potentiation (LTP; Sacchetti et al., 2004; Zhu et al., 2007), as well as an increase of GABAergic signalling onto Purkinje cells from molecular layer interneurons/stellate cells (SC) (Scelfo et al., 2008). Various cerebellar lobules in rodents have been associated with fear learning, but the underlying mechanisms relating to LTP have focused primarily on lobules V/VI. (2) In contrast, NMDAR-mediated long-term depression (LTD) at SC-SC synapses has been associated with extinction (Dubois and Liu, 2021). (3) The medial cerebellar nuclei (MCN) have also been directly associated with fear extinction (Frontera et al., 2020), *via* projections to dopaminergic, GABAergic and glutamatergic neurons in the vlPAG, Vaaga et al. (2020). MCN projections are capable of modulating glutamatergic neurons that express the transcription factor Chx10 in the vlPAG (Vaaga et al., 2020) which have previously been associated with freezing behaviour through their projection to the magnocellular reticular nucleus. This in turn is directly connected to motor neurons of the spinal cord (Tovote et al., 2016). (4) The cerebellar nuclei also project to the VTA; an area associated with the enhancement of fear extinction learning (Carta et al., 2019). (5) Finally, the cerebellum also projects either directly (thalamus, hypothalamus) or indirectly (amygdala, anterior cingulate cortex, hippocampus, striatum) to multiple other areas that could be related to the fear network (Apps and Strata, 2015). PC: Purkinje Cell, MCN: Medial Cerebellar Nuclei, SC: Stellate Cell, GC: Granule Cell, CS: Conditioned Stimulus, US: Unconditioned Stimulus, VTA: Ventral Tegmental Area, vlPAG: VentroLateral PeriAqueductal Grey.

In recent years, there has been a focus on the function of reciprocal connections between the cerebellum and the ventrolateral region of the periaqueductal grey (vlPAG; Whiteside and Snider, 1953; Teune et al., 2000; Koutsikou et al., 2014). The vlPAG is known to play a role in defensive behaviours including freezing (Koutsikou et al., 2014; Tovote et al., 2016) and recent histological studies, have shown in mice that glutamatergic projection neurons from the medial cerebellar nuclei (MCN) make direct connections with GABAergic, glutamatergic and dopaminergic neurons in the vlPAG (see Figure 1; Frontera et al., 2020; Vaaga et al., 2020).

Targeted inhibition or excitation using Designer Receptors Exclusively Activated by Designer Drugs (DREADDs) of the MCN-vlPAG pathway in mice demonstrated that this pathway is involved in extinction of a conditioned response (Frontera et al., 2020). Frontera et al. (2020) showed that inhibition of the MCN-vlPAG pathway during extinction resulted in an increase in freezing behaviour that persisted after the manipulation and during an extinction recall test. When the pathway was activated there was also a smaller, slower, and less pronounced increase of freezing behaviour in comparison to controls which was abolished in extinction recall testing (Frontera et al., 2020). It is unclear why both inhibition and excitation of the pathway using DREADDs resulted in an effect in a similar direction. Temporally targeted optogenetic stimulation of the MCN-vlPAG pathway during delivery of the CS+ tone offset caused a large impairment of conditioned freezing during all the extinction sessions, but did not change extinction recall testing suggesting this modulation was affecting the expression of fear behaviour, but not the memory of extinction itself. Likewise, optogenetic stimulation of the pathway during the pairing of the US foot-shock with the conditioned tone resulted in decreased freezing levels overall during extinction. These data suggest the pathway is involved in supporting the process of extinction or the expression of fear behaviour, but it is unclear whether it contributes to extinction memory.

Further evidence that the cerebellum modulates vlPAG function was demonstrated following pharmacological inactivation of the MCN during consolidation (Lawrenson et al., 2022). Lawrenson et al. (2022) recorded temporally precise single unit activity in the vlPAG in response to the onset and offset of conditioned auditory cue during extinction. Modulation of MCN output during consolidation disrupted the temporal precision at tone offset during early extinction, which was associated with an increase in overall vlPAG unit responsiveness (as measured by response area). This manipulation also resulted in an increase in the duration of freezing epochs in comparison to controls during early extinction, suggesting inhibition of the MCN during fear consolidation affects how freezing behaviour is expressed during recall of the fear memory.

Other experiments targeting the MCN-vlPAG pathway have demonstrated that its modulation during acquisition of the fear memory affects fear-related freezing behaviour during extinction, but does not test extinction directly (Frontera et al., 2020; Lawrenson et al., 2022). These experiments agree that inhibition of the MCN-vlPAG pathway strengthens acquisition of the fear memory, resulting in increased levels of freezing during extinction in comparison with controls, while excitation of the pathway reduces acquisition of the fear memory resulting in decreased freezing during recall of the fear memory and extinction.

Together these experiments (Frontera et al., 2020; Lawrenson et al., 2022) provide evidence that the role of the cerebellum *via* its interactions with the vlPAG is to support fear acquisition, fear memory and the reduction of a cued fear memory to benefit extinction. It is currently unclear whether this pathway contributes to extinction recall and memory of extinguished fear.

## Molecular and physiological mechanisms underlying fear extinction in the cerebellum

In studies using the eyeblink reflex (where a CS tone is paired with an US air puff to the eye), Medina et al. (2002), showed that a disinhibition of the inferior olive prevented extinction of the conditioned response suggesting that both the US during acquisition and lack of US during extinction are encoded by the climbing fibre system, which could in part arise *via* inhibitory input from the cerebellar nuclei. They suggested that the difference between acquisition versus extinction is reflected by climbing fibre signalling relative to background levels of neuronal activity. It is possible that a similar mechanism with a bidirectional modulation of climbing fibres contributes to adaptive regulation of emotional control in the cerebellum.

A number of studies have also investigated the molecular mechanisms underlying fear learning and memory relating to Long-Term Potentiation (LTP) (Sacchetti et al., 2004; Zhu et al., 2007), but very few have investigated extinction. Following fear acquisition in mice, there is an increase in GABAergic signalling from molecular layer interneurons (MLIs) onto Purkinje cells (Scelfo et al., 2008) and neighbouring MLIs in cerebellar lobules V/VI (Dubois and Liu, 2021). This increase in GABAergic signalling between MLIs was abolished in an NMDA-dependent manner during fear extinction, as shown by the deletion of the GluN2D NMDA receptor subunit. Importantly this deletion did not affect acquisition or consolidation but did impair fear extinction (Dubois and Liu, 2021). Blocking GABAergic signalling specifically in granule cells, however, has been shown to cause an increase in general anxiety in mice, especially in females, which also showed decreased interest in social interactions with other female mice, and impaired maternal behaviour (Rudolph et al., 2020). However, this raises the question of whether GABAergic signalling is involved in extinction processes or if it is affecting the general emotional state of the animal.

Overall, current research is lacking a cohesive and integrative understanding of the molecular and physiological mechanisms of cerebellar fear extinction which are needed to support and better understand behavioural findings from rodent and human studies.

## The role of the cerebellum in fear extinction: evidence from human studies

In humans, the cerebellum has been shown to be involved in the acquisition of learned fear (Casey et al., 1994; Ploghaus et al., 1999; Knight et al., 2005; Carlsson et al., 2006; Carlson et al., 2011; Cacciaglia et al., 2013; Michelle Welman et al., 2018), but less is known about its role in fear extinction (Lange et al., 2015). The fMRI studies that report activation in the cerebellum during fear extinction are shown in Table 1. Most sites of activation are in posterolateral regions (Linnman et al., 2012; Faul et al., 2020; Graner et al., 2020; Labrenz et al., 2022), while other studies report activation in the vermis (Kattoor et al., 2014; Utz et al., 2015). These inconsistencies are likely due to differences in experimental design (US type, location and saliency, reinforcement frequency, inclusion of changes of context) and fMRI analysis. For example, Kattoor et al. (2014) were able to detect both posterolateral and vermal differential sites of activation in early, but not late extinction using a visceral US and geometric shapes as conditioned visual stimuli. In comparison, another study also using geometric shapes as the CS, but paired with an electrical stimulation as the US, found only vermal activation in extinction (Utz et al., 2015). A virtual reality study reported activity in lobule VI during late extinction only when CSs (corresponding to different male avatars) were located nearby, and not when presented further away (Faul et al., 2020). Another study using visceral pain as the US reported CS + related activity in early extinction in posterolateral lobules VI, Crus I and Crus II. The CS + related activity was similar to activation during acquisition, and could therefore represent activity related to a remaining fear response (Labrenz et al., 2022).

**TABLE 1:** Studies which support a role of the cerebellum in fear extinction.

Species	Region(s)	Findings	Methods	CS and US types	References
Human	Lobules IV, V, VI, Crus I, Crus II, VIIb, IX, and vermis	Activation in early, but not late extinction	Contrast: CS + > CS−	Visual CS, visceral US	Kattoor et al., 2014
Human	Right lobule VI	Loss of persistent activation for distal, but not near CS + ‘’s during extinction	Contrast: CS + > CS−	Visual CS, electrical US	Faul et al., 2020
Human	Anterior vermis	Extinction learning related activation	Contrast: (CS + _*late*_ > CS−_*late*_) > (CS + _*early*_ > CS−_*early*_)	Visual CS, electrical US	Utz et al., 2015
Human	Lobule VI and Crus I	Extinction learning related activation	Contrast: CS + _*first4*_ > CS + _*last4*_	Visual CS, electrical US	Linnman et al., 2012
Human	Left Crus I	Late acquisition CS + representation changed between early and late extinction	Multivariate representational similarity analysis	Visual CS, electrical US	Graner et al., 2020
Human	Lobule Crus I	Activation related to omissions of the US (extinction)	Contrast: no-US post CS + > no-US post CS−	Visual CS, electrical US	Ernst et al., 2019
	Lobules VI, Crus I, Crus II, and vermis	Activation related to unexpected omissions of the US (acquisition)	Contrast: no-US post CS + > no-US post CS−		
Human	Mean location in lobule VI	Activation related to unexpected omissions of the US (extinction)	Selection of time series for different locations in the cerebellum	Visual CS, heat US	Ploghaus et al., 2000
Human	Lobule Crus II	Activation related to unexpected omissions of the US (acquisition)	Custom fMRI analysis: no-US post CS + > rest	Visual CS, visceral US	Yágüez et al., 2005
Human	Lobules VI, Crus I, Crus II and VIIb	Activation during early extinction	Contrast: CS + > rest	Visual CS, visceral US	Labrenz et al., 2022
Human	Lobules VI, Crus I (mainly)	Reduction of activation from early to late extinction	Contrast: CS + > rest	Visual CS, electrical US	Batsikadze et al., 2022
	Lobules VI, Crus I (mainly)	Activation related to CS prediction during extinction (on a trend level)	Parametric modulation: CS x prediction		
	Lobules VI, Crus I (mainly)	Activation related to no-US prediction error during extinction (on a trend level)	Parametric modulation: no-US x prediction error		
Rodent (mouse)	Vermal lobules V/VI	NMDAR-dependant LTD in stellate cell synapses contributes to extinction	*Ex vivo* slice recording	Auditory CS, electrical US	Dubois and Liu, 2021
Rodent (mouse)	MCN projection to vlPAG	Activation of the MCN-vlPAG pathway during acquisition facilitates extinction	Optogenetic and chemogenetic manipulations	Auditory CS, electrical US	Frontera et al., 2020
Rodent (rat)	MCN projection to vlPAG	Modulation of the MCN during consolidation changes encoding of vlPAG responses during early extinction	*In vivo* electrophysiology	Auditory CS, electrical US	Lawrenson et al., 2022

A brief summary of the key findings is provided for each study, including the cerebellar regions activated, the techniques and analysis applied and the type of CS and US used. Blue rows indicate human studies while orange rows are rodent studies. The human fMRI studies investigate brain activations related to extinction learning by using various contrasts. One common approach is to compare the fMRI signals during the CS + presentation with the CS- (CS + > CS-; Kattoor et al., 2014). This comparison aims to detect any differential activation related to the prediction of the US. It is worth noting that this approach does not fully capture fear extinction learning, as the CS- itself is associated with an absence of the US (i.e., safety learning; Lonsdorf et al., 2017). The reduction of differential activity in a cerebellar region during extinction can suggest that a region might play a role in extinction. Some studies show this reduction directly through a contrast which compares differential activations over time (Utz et al., 2015). Another approach is to directly look at CS + activations without the CS- baseline, although this does not control for non-associative processes (CS + > rest; Labrenz et al., 2022). Other comparisons can be made during the US time window (without US delivery) which may reveal cerebellar activations during partially reinforced acquisition and extinction, and could be related to prediction errors (Ernst et al., 2019). In a recent study (Batsikadze et al., 2022), a deep neural network was trained using skin conductance responses to output prediction and prediction error values. These were then used for parametric modulation in fMRI analysis to directly assess cerebellar activation related to learning parameters. Just three rodent studies are included as very few directly test the cerebellum during fear extinction, where manipulations or observations are made during extinction itself (rather than during fear acquisition or fear consolidation). So far, rodent studies have been limited to the vermis and medial cerebellar nuclei, while human studies primarily show involvement of the posterolateral lobules in fear extinction.

In a more recent fMRI study Ernst et al. (2019) did not find differential CS activation during fear extinction, but did find activation in the US window corresponding to the omission of the US during extinction in left Crus I. Activation was much stronger for repeated unexpected omissions of the US in acquisition, where partial reinforcement allowed the analysis of fMRI responses during CS + no-US windows. As the aversive stimulus is predicted but not received, this activity is hypothesized to be related to prediction error processing which may drive fear extinction learning. During unexpected omissions in acquisition, a differential pattern of activation was again found in left Crus I, but also right Crus I and bilateral lobule VI, Crus II and vermis (Ernst et al., 2019). These observations are to some extent in accordance with another study (Yágüez et al., 2005) where a 50% reinforced phase was followed by a 100% reinforced learning phase. In the 50% reinforced phase, activity during the US window in unreinforced trials resulted in a small but significant cluster in left Crus I. While Yágüez et al. (2005) argue that this activity represents US anticipation without influence of the US presentation, it could instead be interpreted as prediction error related activity due to an unexpected omission of an aversive stimulus.

Different parts of the cerebellum have been reported to match both the unexpected presentation or omission of the US (Ploghaus et al., 2000). In both cases fMRI signals increased indicating an unsigned prediction error, however, activation in response to the unexpected US presentation is likely related to both the stimulus itself and prediction error. This study demonstrated cerebellar activity during the unexpected omission of the US in the initial extinction trial, with mean activities cantered bilaterally around lobule VI. Prediction error related activity during extinction was also found in lobule VI and Crus I on a trend level in a recent study, which used a deep learning model to estimate prediction error values for an fMRI analysis with parametric modulation (Batsikadze et al., 2022). In essence, human fMRI research typically implicates the posterolateral lobules in fear extinction, while the vermis may also play a role, and US window activations suggest an involvement in prediction error processing.

The absence of an expected aversive US results in a better-than-expected outcome, which may be internally perceived and treated as a rewarding event. Consequently, fear extinction learning may be a form of reinforcement learning (Kalisch et al., 2019). In support of this claim, a recent study in mice documented direct excitatory projections from the cerebellar nuclei to the VTA (Carta et al., 2019); a region implicated in the reward circuit (Carta et al., 2019). Dopaminergic (DA) neurons in the VTA are activated following an unexpected US omission during fear extinction (Luo et al., 2018; Salinas-Hernández et al., 2018), specifically during early trials when the prediction error is the highest (Salinas-Hernández et al., 2018). Furthermore, inhibition or excitation of VTA DA neurons at the time of US omission during extinction was, respectively, linked with impairment and enhancement of fear extinction learning. These findings might indicate a role for the cerebellum in reward and reward prediction error processing driving fear extinction.

Until now the role of the human cerebellum in fear extinction has almost exclusively been studied with fMRI in healthy participants. Valuable insights might also be found in patients with cerebellar disease. For example, five patients with lesions of the cerebellar vermis demonstrated a lack of fear conditioned bradycardia, even though no differences were found with matched controls regarding skin conduction responses (Maschke et al., 2002). Additionally, non-invasive brain stimulation could provide a method for altering cerebellar activity during fear extinction (Klaus and Schutter, 2022), and test fMRI observations. For instance, transcranial direct current stimulation (tDCS) applied over the right dorsolateral prefrontal cortex before and during extinction was found to change connectivity in the anterior lobe of the cerebellum and lobule VI (Ganho-Ávila et al., 2022). However, likely due to the highly convoluted cerebellar cortex the effects of cerebellar tDCS have been difficult to reproduce in cerebellar-dependent learning paradigms, such as eyeblink conditioning and reach adaptation (Kimpel et al., 2020). Attention instead has moved to the possibility of using other non-invasive stimulation techniques such as transcranial focused ultrasound neuromodulation to activate the cerebellum. This method has shown over the last years promising results in reaching deep brain structures in humans and mice by enabling higher spatial resolution modulation (di Biase et al., 2019). Combining fMRI and EEG can also provide increased temporal resolution to study the cerebellum and its role in the timing of cues in the fear network (Warbrick, 2022).

## Linking rodent and human cerebellar research in fear extinction

There are a number of important differences between rodent and human cerebellar research regarding fear extinction that currently make it difficult to directly compare results across species. Rodent studies have primarily used males, whereas human studies involve both sexes. Indeed, growing evidence indicates that anxiety disorders are prevalent in females (McLean et al., 2011) and that sex hormones have an effect on fear extinction processes (Graham and Milad, 2013; Merz et al., 2018), making it central to consider both sexes. In rodents, the US signal is most commonly presented in the form of an electrical shock while in humans the US signals are more diverse, including the use of sounds (scream or loud noise), visceral pain or olfactory cues (rotten egg smell), as well as electrical shocks. Furthermore, it is likely that in rodents the US is perceived as a real threat, while in humans it is perceived as an artificial experiment. In the experiments described above, the CS in rodents is an auditory signal (which can be variable in frequency, duration and tone type). In humans the CS’s used are predominantly visual, while auditory cues such as tones can also be used. This variability in US and CS signals between rodents and humans, as well as across studies within the same species, needs to be addressed in order to make studies more translatable and reproducible. Behavioural measures of defensive state in rodents (e.g., freezing) are not directly comparable with autonomic measures of fear in humans (e.g., skin conductance responses). Moreover, rodent research typically involves targeted manipulation of specific cerebellar regions (e.g., cerebellar vermis and MCN) or neuronal types, that allows investigation of distinct neural pathways, but lacks a comprehensive overview of all potential cerebellar areas and projections involved. In humans, fMRI allows for observation of the whole cerebellar cortex during fear extinction, however, focused imaging of the human cerebellar nuclei and its outputs is lacking. Often the strongest fMRI signal is observed in the lateral hemispheres, while in rodents the focus has been on the vermis and MCN meaning a direct comparison is not possible. To date, rodent fMRI studies during fear learning have excluded the cerebellum (Brydges et al., 2013; Harris et al., 2015), and so its future inclusion is essential to better understand the cerebellar role in fear extinction.

## Discussion

In summary, there is a range of anatomical, physiological, behavioural, and imaging evidence that the cerebellum plays a role in the extinction of conditioned defensive states in rodents and humans. Taking into consideration the multiple connections of the cerebellum with the fear network (Apps and Strata, 2015; Tovote et al., 2015) and the role of the cerebellum in fear learning and extinction processing (Frontera et al., 2020; Lawrenson et al., 2022), its overall contribution is likely to coordinate different aspects of fear circuitry in order to facilitate the temporally appropriate adaptive responses of the organism towards changes in the environment. There are a number of key unknowns highlighted in this review. In particular, current research is lacking a basic understanding of the molecular and physiological mechanisms underlying cerebellar fear extinction processes. In rodent and human studies, further fear extinction experiments are also needed to better understand the role of cerebellar projections with other key regions in the fear network such as the VTA in reward/safety signalling. It is also unclear how different cerebellar regions in both rodents and humans contribute to the emotional, motor, sensory and autonomic components of fear extinction, and how this changes with task specificity. Do individual differences in the way the cerebellum processes fear learning and extinction contribute to anxiety disorders? Given the prevalence of anxiety disorders in women, it is also unknown whether there are sex differences relating to cerebellar function. Furthermore, the question remains, how can we better link human and rodent studies to make data more comparable so that rodent studies are more clinically relevant?

## Author contributions

FS-S, VS, EN, and AD collaborated equally on the preparation of the manuscript. FS-S wrote the sections on molecular studies in rodents and created Figure 1, while also contributing to the discussion. VS focused on other rodent-related sections and helped with the introduction. EN wrote the portions on human fMRI studies, created the table, and contributed to the discussion. AD wrote other human-related parts of the review and contributed to the introduction. CL supervised review, provided the correction, and revisions. DT and RA gave feedback and proofreading. All authors contributed to the design and conceptualization of the review and approved the final submitted version.
