# Corrigendum: The cerebellum and fear extinction: evidence from rodent and human studies

**DOI:** 10.3389/fnsys.2024.1488334

**Published:** 2024-09-13

**Authors:** Alice Doubliez, Enzo Nio, Fernando Senovilla-Sanz, Vasiliki Spatharioti, Richard Apps, Dagmar Timmann, Charlotte L. Lawrenson

**Affiliations:** ^1^Department of Neurology, Center for Translational Neuro- and Behavioral Sciences (C-TNBS), Essen University Hospital, University of Duisburg-Essen, Essen, Germany; ^2^School of Physiology, Pharmacology and Neuroscience, University of Bristol, Bristol, United Kingdom

**Keywords:** cerebellum, fear extinction, fMRI, prediction error, fear behaviour, cerebro-cerebellar circuits, electrophysiology

In the published article, there was an error in the Funding statement. The MRC funder grant number was incorrect. The project number listed was “MR/019484/1” when it should have been “MR/T019484/1”. The correct Funding statement appears below.

